# Delay Optimal Schemes for Internet of Things Applications in Heterogeneous Edge Cloud Computing Networks

**DOI:** 10.3390/s22165937

**Published:** 2022-08-09

**Authors:** Abdullah Lakhan, Mazin Abed Mohammed, Karrar Hameed Abdulkareem, Mustafa Musa Jaber, Jan Nedoma, Radek Martinek, Petr Zmij

**Affiliations:** 1 Department of Cybersecurity and Computer Science, Dawood University of Engineering and Technology, Karachi City 74800, Sindh, Pakistan; 2 Institute of Artificial intelligence and Blockchain, Guangzhou University, Waihuan West Road, University Town, Guangzhou 510006, China; 3 College of Computer Science and Information Technology, University of Anbar, Anbar 31001, Iraq; 4 College of Agriculture, Al-Muthanna University, Samawah 66001, Iraq; 5 College of Engineering, University of Warith Al-Anbiyaa, Karbala 56001, Iraq; 6 Department of Computer Science, Dijlah University College, Baghdad 00964, Iraq; 7 Department of Medical Instruments Engineering Techniques, Al-Farahidi University, Baghdad 10021, Iraq; 8 Department of Telecommunications, VSB-Technical University of Ostrava, 708 00 Ostrava, Czech Republic; 9 Department of Cybernetics and Biomedical Engineering, VSB-Technical University of Ostrava, 708 00 Ostrava, Czech Republic; 10 Industrial Engineering—Brose Group, Prumyslovy Park 302, 742 21 Koprivnice, Czech Republic

**Keywords:** JTOS, CLIP, SDN, task scheduling, framework, dynamic environment

## Abstract

Over the last decade, the usage of Internet of Things (IoT) enabled applications, such as healthcare, intelligent vehicles, and smart homes, has increased progressively. These IoT applications generate delayed- sensitive data and requires quick resources for execution. Recently, software-defined networks (SDN) offer an edge computing paradigm (e.g., fog computing) to run these applications with minimum end-to-end delays. Offloading and scheduling are promising schemes of edge computing to run delay-sensitive IoT applications while satisfying their requirements. However, in the dynamic environment, existing offloading and scheduling techniques are not ideal and decrease the performance of such applications. This article formulates joint and scheduling problems into combinatorial integer linear programming (CILP). We propose a joint task offloading and scheduling (JTOS) framework based on the problem. JTOS consists of task offloading, sequencing, scheduling, searching, and failure components. The study’s goal is to minimize the hybrid delay of all applications. The performance evaluation shows that JTOS outperforms all existing baseline methods in hybrid delay for all applications in the dynamic environment. The performance evaluation shows that JTOS reduces the processing delay by 39% and the communication delay by 35% for IoT applications compared to existing schemes.

## 1. Introduction

These days, the usage of industrial automation applications in the Internet of Things (IoT) enabled paradigm has been growing progressively in practice. Industrial automation applications are smart homes, smart agriculture, smart healthcare, and smart transport with different data analytics sensors that offload their data to the cloud server for execution. Recently, Internet of Things (IoT) applications, such as healthcare, autonomous vehicles, and smart homes, are increasing progressively. IoT brings the efficient resource environment for the industrial automation applications with the collaboration of fog and cloud networks. With millions of sensors and intelligent devices, various applications can be developed that generate vast amounts of data and have stringent latency requirements. These applications include smart grids, innovative healthcare, intelligent vehicles, intelligent buildings, and many more. Typically, in cloud computing, data are sent to remote data centers for computing and storage. However, with the advent of IoT platforms, novel applications and use cases have emerged with bandwidth and latency requirements that cannot be met by traditional cloud computing. Hence, a new computing paradigm was created to cater to applications with low latency requirements. Fog computing extends cloud computing by bringing the storage and computing facility to the edge of the network, reducing bandwidth requirement and latency. Fog computing, also called edge computing, does not replace cloud computing. Fog nodes are used for short-term analytics with limited data. Resource-intensive computing and long-term analytics take place at cloud data centers. The Open Fog Consortium (OFC) is an open-source multi-vendors hybrid architecture that allows the IoT applications that leverage fog and cloud architectures mutually [1].

Core mobile cloud computing (MCC) offers unlimited shareable services to the users [2]. However, it is often incurred with long end-to-end latency due to the multiple hops away from users. Fog computing is a subset of cloud computing that enables cloud services at the edge of users’ networks with ultra-low latency [3]. Typically, IoT sensors and devices are resource constrained and generate huge amounts of data that are processed by applications [4]. IoT-based applications comprise various tasks that can be computed or data-intensive. Due to the high demand for computing and other resources, compute-intensive tasks are offloaded. Task offloading is a method which transfers all compute-intensive parts of an application either to the fog or public cloud for processing [5].

The major challenge in task offloading is to decide whether the task is to be sent to the fog or cloud for processing [6]. Though the decision is based on several factors, bandwidth, data, and latency remain the most critical. Further, the heterogeneous nature of IoT applications’ data, along with the high quality of service (QoS) requirements in terms of latency and efficiency, makes the decision of task offloading more complex [7]. In order to meet latency requirements, the tasks are offloaded jointly in heterogeneous computing nodes to solve scheduling. Task offloading problems for IoT applications have been widely addressed separately by many researchers. For instance, these studies [8,9,10,11,12,13,14] investigated the delay optimal offloading problem in the mobile edge cloud (MEC). The aim is to reduce device energy consumption and accelerate application performance on resource-constrained devices. Furthermore, the authors [15,16,17,18] addressed the issue of task scheduling in the MEC. The goal is to schedule edge cloud resources to improve application performance and decrease the energy consumption of cloud nodes. The joint optimization problem has been investigated by these studies [6,7] to address task offloading and resource allocation problems mutually for IoT applications. However, the work mentioned earlier did not focus on task offloading and task scheduling problems jointly to meet the requirements of latency-sensitive tasks in the fog cloud networks. Therefore, there is a need for a framework that can optimize IoT application performance by considering both task offloading and scheduling problems in fog cloud networks.

This article formulates delay-efficient joint offloading and scheduling as combinatorial integer linear programming (CLIP) problems in heterogeneous fog cloud networks for industrial automation applications. All the problem constraints are linear integers and objective functions to be minimized for each task. The industrial automation applications based on IoT are smart homes, augmented reality (A.G), E-Business (E-Factories), and E-Healthcare; they offload their workloads to the fog cloud for the processing in the system. Furthermore, heterogeneous fog cloud networks are the combination of different capacity fog computing and core cloud computing. This research aims to minimize the total delays (i.e., network delay and cloud delay) of a task during offloading and scheduling. Each IoT application consists of independent tasks. Each task includes workload attributes and deadline constraints. The fog clouds are dispersed and connected with users via the base station (BS). All BSs are linked via switches and managed by a software-defined network (SDN) control [8].

The considerations in our article are quite different from the existing ones in several aspects:

- Generally, former studies [7,8,9,10,11,12,13,14] made offloading decisions based on single criteria, such as either mobile battery power threshold or application total time limitation. Whereas, in a dynamic environment, a single threshold value-based offloading decision is not accurate; thus, we consider the multi-criteria task offloading-based decisions more accurate, and they incur lower overhead during the task offloading decision.
- Existing task scheduling problems generally involve soft deadlines in the homogeneous edge only or core cloud-only resources [15,16,17,18,19,20,21,22,23]. Nonetheless, the frameworks proposed in those research works consider mutually heterogeneous fog cloud resources for IoT applications. Furthermore, each task has a hard deadline and must meet the stringent latency requirements during offloading and scheduling.
- The high-level placement policy of the proposed fog-cloud architecture for real-time and delay-sensitive applications is different from earlier works’ architectures regarding offloading decisions and task prioritizing for scheduling.

The state of the art formulated the network delay and computational delay for IoT tasks. The key objective is to minimize the delay of tasks in the system. There are many types of delays in the fog cloud network. For instance, network delay, communication delay, wait for delay, and processing delay. However, these studies only considered static offloading and static scheduling in their solutions without considering the mobility factors and dynamic aspects of functions in the system. Therefore, real-time, multi-parameter aware offloading and scheduling in dynamic environments are widely ignored in the state-of-the-art studies. This study focuses on two types for each task, such as network delay and computational delay. The network will optimize by the offloading technique, and computational delay will optimize by dynamic scheduling in the study.

The Major contributions of this article are summarized below:

1 The problem considered the hybrid delay, a combination of network delay and computation delay under a dynamic environment where transient failure in resources always occurs. This study suggests an architecture that shows how to solve the joint offloading and scheduling problem in different steps. The study considered the following steps: offloading, sequencing, scheduling, and transient failure awareness for IoT applications. The architecture aims to construct an environment to facilitate applications to run with distributed resources in the network. The architecture components or steps are discussed in the proposed solution in detail.
2 The fuzzy multi-criteria task offloading method is proposed, which makes an optimal offloading decision that adopts changes during the offloading decision process, ensuring that the network context changes do not degrade the task offloading performance.
3 The work devises latency efficient task sequence in which their competent order arranges all tasks, thereby meeting all application requirements.
4 The task scheduling method with topological sorting, searching, and transient failure methods proposed by the study to deal with the robustness of the applications during scheduling in the network.

This study formulated the CLIP problem for IoT, which is different from existing studies in the following way. This work formulated the joint optimization as the CLIP problem, which is a well-known NP-Hard problem. To solve the joint optimization problem, the JTOS framework is proposed. For offloading and scheduling, the prediction of delay and QoS of applications are satisfied during the process. We consider the round-trip delay between users and BS and BS to computing nodes in the network delay. Existing studies only considered either user to node delay or user to BS delay. This study introduced a novel transient failure method, which can handle any transient failure tasks during the process. The previous works focused on the failure of tasks based on checkpointing and or primary backup method. These methods cannot handle transient failure and consume much more resources of the nodes during the recovery of tasks from the failure state.

The rest of the article is organized as follows. Section 2 elaborates related work, followed by Section 3, which describes the proposed description and formalizes the problem under study. A heuristic is proposed for the considered problem in Section 4 that describes the proposed algorithm: JTOS. Section 5 discusses the experiments and results, whereas Section 6 concludes the article.

## 2. Related Work

Recently, the usage of IoT with different computing nodes (e.g., fog/edge computing) is increasing day by day [24]. Many partitioning, offloading, and scheduling frameworks, architectures, and methods have been suggested to improve the energy, cost, and delay of applications and solve the CLIP problem. In the literature, many efforts have been made to solve the different IoT application problems. We analyze the actions of existing studies in the table to solve the CLIP problem (Table 1).

Debashis et al. [1] suggested a multi-leveling offloading method to solve the partitioning and offloading problem in distributed mobile cloud architecture. The goal was to minimize the power and latency of the application during the process in the architecture. The study considered the single parameter offloading (e.g., battery energy/delay), static offloading, network profiling technology, and fixed resources (e.g., mobile and cloud resources) and solved as integer linear programming. However, due to the long distance between mobile users and the cloud, the offloading faced end-to-end latency issues during the process. Shahryari et al. [2] investigated energy-delay-aware offloading for latency-sensitive applications. The study formulated this problem as linear programming with non-linear constraints. The single parameter offloading (e.g., battery energy/delay), static offloading, program technology, and fixed resources (e.g., mobile and fog resources) have been considered. However, due to the high ratio of user requests, the resource-constraint fog node faced an overloading situation during the offloading process. Aruba et al. [3] formulated a scheduling convex optimization problem for the IoT in a fog cloud network to minimize the lateness of the applications. The study considered two types of workload, including latency-sensitive and delay-tolerant with multi-parameters (e.g., workload size and offloading time) in the static resource environment. The study presented the window algorithm for application profiling which may decide whether the workload of the application offloads or not in the distributed fog cloud network. Lin and Fan et al. [4,5] suggested a SDN-based fog cloud network for IoT applications. The offloading and resource allocation-aware schemes are widely suggested to minimize the end-to-end delay of applications. The combinatorial optimization (e.g., Concave) and linear programming (e.g., Quadratic) based objectives were optimized. The multi-parameters (e.g., local execution, network execution, and computing execution delay were taken into consideration) during offloading and resource allocation. The network changes are also considered an adaptive environment where offloading is performed based on new available values instead of old network contents.

The authors in [6,7,8,9,10] investigated joint offloading and resource placement problems for IoT applications in distributed fog/cloudlet cloud networks. These studies considered the dynamic and adaptive environment where network contents and resource placement can change at runtime. The proposed scheduler and offloader engine adopt any runtime changes during the initial process. Based on the experience, a new offloading decision will be more optimal than the existing one. The offloading decision is dynamic concerning resource placement and offloading, where remote procedure call methods are formulated as the integer constraints and the quadratic problem. The objective function was a convex function, i.e., minimize delay, and all variables are convex linear integers in the considered problem.

The authors in [8,11,12,13,14] investigated resource provisioning and cost-aware offloading and scheduling problems for IoT applications in the distributed mobile fog cloud network. The SDN controller was implemented to facilitate mobility features of applications during roaming among networks. The dynamic environment and hybrid static and dynamic offloading decisions were taken into consideration. The resource cost, budget, rent and application delay, and energy objectives were optimized as joint linear integer programming optimization problems. All coarse-grained workloads are scheduled under their maximum threshold level to avoid any violence of users. The authors in [15,16,17,18,19] suggested that frameworks and architectures solve deadline-aware offloading and scheduling problems for IoT applications. They formulated problems as integer linear programming where all objective functions and constraints are linear and integer variables. The budget, energy, renting cost, scheduling cost, and offloading delay objectives are optimized via different optimization methods. The orchestrator controller (e.g., SDN and system components) are implemented to offer mobility-aware services to the IoT vehicle applications during their roaming features. The dynamic offloading decision (e.g., static and dynamic) and dynamic workload assignment in fog cloud network were considered during the problem formulation. Table 1 shows the efforts of existing studies in the area.

Furthermore, the authors in [1,3,5,8,11] proposed optimization algorithms based on a genetic algorithm (GA) with many components (e.g., resource searching, resource allocation and offloading) for IoT applications. The suggested that mixture architectures are based on fog-cloud nodes with virtual machine implementation to serve users’ requests. The proposed methods contained average time complexity during the process of applications in the network. The linear and global searches are parts of GA during the execution of applications. The works [2,4,6,10,12] devised optimization techniques based on particle swarm optimization (PSO) meta-heuristics with many components (e.g., resource searching, composition, allocation, and migration). The fog-cloud was implemented with virtual machines to process the workload of applications with their constraints. To obtain the complexity of the algorithms, the lightweight and linear search-based iterative model was devised by studies. The studies [13,14,15,16,17,18,19] devised different optimization heuristics based on Bundle branch-bound, Subgradient linear search, Interior-Points, and Cutting-plane Hungarian with different types of searching methods. These methods search for the best resource among edge/fog cloud nodes before allocating tasks to them. The time complexity is lower because these studies implemented linear search, where all optimal solutions select randomly based on integer parameters.

Recently, the complexities of IoT applications are increasing day by day. For instance, healthcare applications contain real-time tasks, which require continuous attention from network and computation nodes. Due to the uncertainty of the network due to mobility and traffic, the ratio of failure tasks, delay, and deadline can occur widely. The uncertainty and fluctuation in computing nodes can lead to the violence of the quality of service of applications in the system. Therefore, all existing conventional heuristics cannot adapt to any dynamic changes and do not support the complex requirements of applications. Recently, dynamic environment aware deep reinforcement learning (DRL) aware heuristics were proposed [20,21,22,23,25,26,27] to solve the complex IoT problems. The DRL approaches can work better in a dynamic environment via different states where all states are independent. These approaches are achieving long-term goals in terms of offloading and resource allocations. However, many issues remain in both conventional methods and machine learning approaches when solving the joint offloading and scheduling problem for IoT applications.

## 3. Proposed Solution

**Figure 1:**
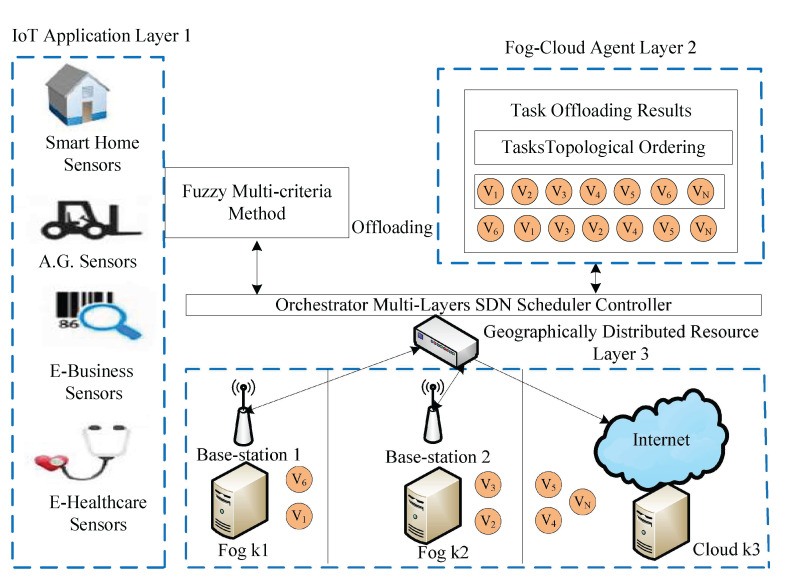
Joint Hybrid Delay Optimal Offloading and Task Scheduling for IoT Applications in Fog-Cloud Architecture.

This section aims to discuss the importance and all steps of the proposed architecture. As mentioned above, this study considers the joint offloading and scheduling optimization problem as a CLIP for IoT applications in the distributed network. The proposed architecture consists of three main layers: IoT application layer, agent layer, and resource layer, as shown in Figure 1. All layers are managed and controlled by the agent layer.

The application layer consists of different applications, where each application is composed of various independent tasks. However, resource-constrained local devices (e.g., limited battery, computational capability, and storage) cannot run all tasks locally on devices. Therefore, a Fuzzy multi-criteria method (FMCM) is implemented at the application layer, which boosts the performance of all applications via an offloading process based on QoS requirements (e.g., deadline and hybrid delay). Initially, FMCM generates an offloading result based on QoS requirements and sends it to the agent layer. Then, the agent layer allows devices to offload their tasks to the system for efficient execution.

The agent layer accepts offloaded tasks based on their offloading result for further execution. Initially, all tasks are sorted into topological order based on their deadlines and total delay. The main reason behind sorting is that all tasks have different sizes, deadlines, and resource requirements. Therefore, delay-sensitive tasks with the lowest deadline must have high priority compared to delay-tolerant tasks with long deadlines. Furthermore, based on sorting order, all tasks are scheduled onto different computing nodes based on their offloading results. In the dynamic environment, in different timezones, the ratio of user traffic is different; therefore, due to the ingests percentages of users in peak hours (8 am to 4 pm), the transient failure of resources often occurs in the system. The transient failure aware (e.g., detection, retry, and familiar failure schemes) handles all transient failure tasks and runs all applications robustly without losing their generosity. The orchestrator, multi-layers SDN scheduler controller is the primary controller in the agent layer, responsible for managing load balancing and connectivity of all computing nodes and all layers, and it helps to monitor resource status in the network.

The resource layer combination of fog nodes and cloud nodes is connected with the SDN controller and BSs. Here, the SDN controller allows devices to make the offloading request to the associated BSs and ensures that BSs are directly connected with computing nodes for further processing. For instance, BS 1 was directly connected with the fog node $k_{1}$ to process task $v_{1}$ and $v_{6}$. Whereas BS 2 connected with fog $k_{2}$ and processed the tasks $v_{3}$ and $v_{2}$. The delay-tolerant tasks are scheduled on fog nodes; however, all delay-tolerant tasks must be scheduled on cloud computing for efficient processing. For instance, $v_{5}$ and $v_{4}$ and the rest of the tasks are offloaded to the remote cloud by the SDN controller for execution via the Internet.

### 3.1. System Model Scenario

As we mentioned above, the study considered leaving dynamic requests of users in the system. Therefore, resources, network channel capacity, and QoS (quality of service) and quality of experience (QoE) requirements of applications are dynamic. The scenario of IoT applications in the fog cloud network is defined as follows. Initially, all IoT applications (e.g., mobile devices, sensors, cameras, vehicles, etc.) are connected to the BSs. Each application is only connected with one BS at a time. In comparison, many base stations are connected with fog nodes which are dynamically distributed and managed by the SDN control plane. All the BSs are connected with fog nodes via different fiber optics switches. The SDN control plane is also associated with the remote cloud via wire Internet to process the delay-tolerant tasks of applications during execution. All BSs and fog nodes are resource constraints; therefore, SDN control is also responsible for load balancing among BSs and computing nodes.

### 3.2. Problem Formulation

The architecture leverages three different core technologies: IoT sensor-based technologies, wireless technologies (i.e., WiFi, Bluetooth, and cellular network), and computing frameworks (e.g., fog node and cloud node). The considered joint optimization task is offloading and the scheduling problem for IoT applications incurred two kinds of delays: network delay and computation delay. Thus, total delays for each task have network delay and computation delay during offloading and execution. The notation of the study is described in Table 2.

### 3.3. Dynamic Environment

The problem constraints, such as computing resources, network contexts, and task size, are highly dynamic in the study. This work considered the in and out user requests in the system as a dynamic environment without mobility features. Although the mobility feature is a part of the dynamic environment, this study did not consider the mobility of the application in the current version of the work.

### 3.4. Network Delay

The network delay for IoT applications is comprised of round-trip delays, such as the delay between a task $v_{i}$ and associated base station *b* and *k*th computing node. The notation $v_{i}↔b$ shows round-trip delay between task $v_{i}$ and assigned BS *b*. Whereas the notation b↔k shows round-trip delay between the base station *b* and computing node *k*. Each application is composed of different types of tasks, such as video, audio, image, text, and so on. However, it is not trivial to know in advance what amount of task data will be carried via a channel per second or at what rate the channel transfers task data to the cloud for execution. There are many factors that can interrupt communication links during task offloading and downloading, such as noise, inference, and intermittency in the wireless network [28]. Thus, our task offloading decision method adopts dynamic changes of network contexts during offloading. The binary variable y={0,1} shows either the offloaded task *i* being implied in base station *b* in the coverage area $y_{ib}=1$ or not $y_{ib}=0$. The communication delay for a task during offloading and downloading between BS and computing node can be estimated in the following way:

(1) $$T_{i}^{net}=y_{ib}\left(v_{i}↔b+b↔k\right)\times C.$$

Equation (1) determines the maximum capacity *C* of the channel and measures the network delay of each task, which is the sum of delay between the user and BS and BS to any particular computing node during offloading and downloading. In simple terms, it is round-trip delay between a user to BS and BS to computing node.

(2) $$rc=\left(\frac{W_{i}}{UB\cdot wc}+\frac{W_{i}^{\prime }}{DB\cdot wc}\right)log_{2}\left(1+\frac{S}{N}\right).$$

In Equation (2), the variable rc determines the usage ratio of channel resources. Where UB.wc is the uploading bandwidth (i.e., not fixed) of the wireless channel, wc determines the hertz (H), and UB.wc is the downloading bandwidth. The variable *S* is the signal power of the network in watts, and *N* is the present noise and inference in the wireless during task offloading to the fog cloud network. Whereas the variable $log_{2}\left(\frac{W_{i}}{UB\cdot wc}\right)+\left(\frac{W_{i}^{\prime }}{UB\cdot wc}\right)$ is the amount of data offloaded and downloaded to and from BS and computing. The network delay of all tasks between all BSs and computing nodes is determined in the following way.

(3) $$\begin{array}{cc} T^{net}= & \sum_{G=1}^{A}\sum_{i=1}^{N}y_{ib}^{G}\left(v_{i}^{G}↔b+b↔k\right),\\ \; & b\in B\;,\;k\in M. \end{array}$$

Equation (3) calculates the network delay of all tasks during offloading and downloading.

#### Computation Delay

The aim of the study is to offload and schedule all independent tasks of different IoT applications to the heterogeneous computing nodes k∈M to reduce the maximum delay of all tasks. The tasks are represented by $\left\{v_{1},v_{2},v_{3},\cdots ,v_{N}\right\}$. Each task $v_{i}$ has workload $W_{i}\left\{v_{i}=1,\cdots ,N\right\}$ and deadline $d_{i}$, which is defined by the user when tasks are offloaded to the proper computing node. All computing nodes are heterogeneous and represented by $\left\{k_{1},k_{2},\cdots ,k_{M}\right\}$. Each computing node has a different computing speed which is depicted as $\zeta_{k}$, where k=1,2,⋯M. We denote the computing resources of all computing nodes in this way, i.e., $\epsilon_{k}=\left\{k=1,\cdots ,kM\right\}$. The notation $\epsilon_{k}$ denotes particular resources of the node *k*. To reduce the computation delay of all submitted tasks, we assign each $v_{i}$ to the ideal computing node to meet the deadline constraint of all tasks with minimum delay. Meanwhile, a task $v_{i}$ is executed by a single cloud *k*. The decision variable is employed $x_{ik}$ and either task $v_{i}$ is assigned to *k* or not. The execution time of the task $v_{i}$ on *k*th computing node can be estimated as given in Equation (4). The computation delay on a particular node of a task *i* is determined in the following way:

(4) $$T_{i}^{e}=x_{ij}\frac{W_{i}}{\zeta_{k}}.$$

Equation (4) measures the execution delay of a task on the particular computing node. Similarly, the execution delay of all tasks on all computing nodes is determined in the following way.

(5) $$T^{e}=\sum_{G=1}^{A}\sum_{i=1}^{N}x_{ij}^{G}\;\frac{{W_{i}}^{G}}{\zeta_{k}}\;where\;k\in M$$

Equation (5) calculates the computation delay of all tasks on heterogeneous computing nodes.

The considered problem is mathematically modeled as below:

(6) $$T_{total}=T^{net}+T^{e}.$$

whereas $T_{total}$ is the total delay (e.g, communication delay and computation delay) of tasks of all applications in distributed computing nodes as determined in Equation (6). The objective function is to minimize communication delay and computation delay for each task.

(7) $$\min T_{total}.$$

The considered problem is a convex linear integer optimization problem, where Equation (7) is a convex main function and computation delay and network delay are the convex constraints in the problem.

(8) $$rs=W_{i}\leq \sum_{k=1}^{M}\epsilon_{k},\;\forall \left\{i=1,\cdots ,GN\right\}\forall A.$$

The requested workloads of tasks must not be exceeded by the limit of resource capacity that is ensured in Equation (8). Whereas rs is an integer variable that shows the 0 and 1 status, if it is greater than zero, it means nodes have sufficient resources to process the workload. Otherwise, it shows 0.

(9) $$F_{i}=B_{i}+T_{i}^{e},\;\forall \left\{i=1,\cdots ,GN\right\}\forall A.$$

The finish time ensures that all tasks are executed under their deadlines with a minimum lateness determined in Equation (9), whereas $B_{i}$ is the beginning time of a task.

(10) $$B_{i}=1-T_{i}^{e},\;\forall \left\{i=1,\cdots ,GN\in A\right\}.$$

The beginning time of a task on the same machine is equal to the execution of the current task during scheduling, as determined in Equation (10).

(11) rc≤C,∀{b=1,⋯,B}.

Each network channel has limited capacity to offload workloads from users to computing nodes; therefore, Equation (11) ensures that the capacity of channel is sufficient for offloading all workloads to the system.

(12) $$\sum_{G=1}^{A}\sum_{i=1}^{N}x_{ik}=1,\;\forall \left\{k=1,\cdots M\right\}.$$

Each task can only assign to one computing node, as defined in Equation (12).

(13) $$\sum_{k=1}^{M}x_{ik}=1,\;\forall \left\{i=1,\cdots ,GN\right\}\forall A.$$

Each computing node can only execute one task at a time, as defined in Equation (13).

(14) $$\sum_{b=1}^{B}y_{ib}=1,b\in B.$$

Each task can access one base station at a time. It depends upon the availability of the network; therefore, the binary variable $y_{ib}$ shows 1 if a task accesses the particular base station, otherwise it is zero, as shown in the Equation (14).

(15) $$x_{ij}\in \left\{0,1\right\},y_{ib}\in \left\{0,1\right\}.$$

Equation (15) shows that task *i* is either assigned to the computing node *k* or not.

The combinatorial Integer Linear Programming (CLIP) offers an optimization solution to the linear problem. The offloading and scheduling are linear and have a trade-off between network delay and computational delay in the considered problem. CLIP has an objective function with different constraints. Therefore, communication delay and computation delay for each task are determined based on their primitives. For instance, data size required resources to execute data and make the deadline. Mainly, the performance of the objective function depends upon available resources, including network resources and computation resources. The formulation of CLIP is performed based on Equations (1)–(14).

## 4. Proposed Algorithmic Jtos Framework

The study considered offloading and scheduling problems as a joint optimization and formulated a combinatorial integer linear programming (CILP). The objective function is an integer value, where all constraints are integer numbers and are denoted as a convex set. For a feasible solution, it is necessary to satisfy all conditions of the problem during the entire process in the system. The CILP is an NP-hard problem when it processes heterogeneous machines in the distributed fog cloud network. In joint optimization, offloading decides whether to offload or not based on certain values to obtain the minimum network delay and computation delay of applications. Moreover, scheduling will handle resource allocation mechanisms of tasks under deadline and failure constraints. Keep the balance between the total delay of $T_{total}$ and deadline and constraints, and the joint optimization will achieve the overall objective of the study. Furthermore, the CILP problem will be divided into more sub-problems, such as offloading, sequencing, and scheduling. To solve the CILP problem, the study proposes the JTOS algorithmic framework, which consists of different components for processing user requests. JTOS framework initially takes the input of all tasks of applications. The study suggests that an Algorithm 1 is the main algorithmic framework that consists of different methods in the sequences. For instance, the FMCM method is the framework method that makes the offloading decision based on the following parameters (e.g., network delay, computation delay, required computing instructions (ms) data size, and total delay). The Fuzzy indexes and weight ratios consist of different attributes, including execution time, communication time, resource availability, and deadline. The pairwise comparisons of giving elements are produced based on the normalized comparison scale on nine levels as illustrated in Table 3. Task offloading results are a result of tasks, as shown in Table 4. Each task has different requirements, such as a small workload, a small deadline, and being delay-sensitive and delay-tolerant. Therefore, all listed tasks are furthermore sorted into the proposed topological ordering of their needs. Based on topological ordering, machine learning-based search finds the optimal computing node for each task. Based on topological sorting, all tasks are scheduled onto search nodes based on their objective function. However, initial scheduling incurs the failure of tasks it will handle in two ways. Firstly, the transient failure tasks will recover under their deadlines and comprehensively failed tasks will re-offload from scratch to the system. The work discusses all components in the corresponding subsections.
**Algorithm 1:** JTOS Framework
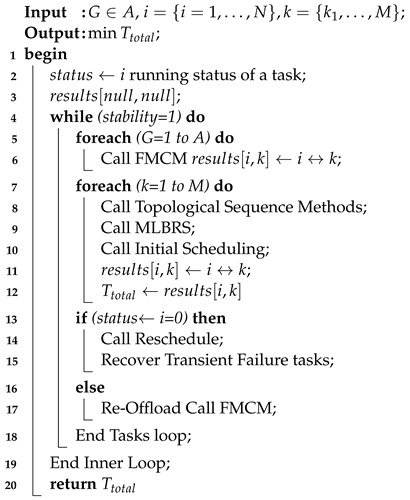


### 4.1. Fuzzy Multi-Criteria Method (Fmcm)

The offloading is the sub-problem of the CILP type problem, which makes the decision when and where to offload tasks in such a way that total $T_{total}$ is minimized for all applications. The offloading problem only ensures that it delays optimal offloading from users to computing nodes without considering deadlines and failure situations of tasks. In this study, offloading is a multi-criteria decision problem, where decision parameters have some weights to make the offloading for tasks. The fuzzy logic algorithm accommodates solving an enigma input. This study presented the FMCM offloading method for the applications, where the FMCM method evaluates the rank of each criterion according to criterion, i.e., ω based on Equation (15). The goal is to sort and determine all ranks based on their given requirements. Furthermore, the FMCM method normalized aggregated fuzzy importance weight for each criterion based on Equation (16). After that, the technique normalized the matrix for all applications G∈A for each measure based on Equation (17). Similarly, the FMCM method normalized stored weights based on Equation (18). FMCM constructs the weighted normalized fuzzy decision matrix of application and makes decisions in the fifth and sixth. The seventh FMCM determines the fuzzy positive and negative ideal solution based on Equations (19) and (20). In the final step, FMCM determines the fuzzy closeness computing node for each task and ranks the alternatives according to their closeness based on Equation (21). To solve the multi-criteria offloading decision, we propose the FMCM. The FMCM determines a decision based on the given weights to the criteria of the task during offloading matrix, i.e., $G\{v_{1},v_{2}\cdots ,v_{N}\}$, where each attribute includes relative weight for their importance, i.e., ω={0.1,0.4,0.3,0.5}. We formulate the task offloading problem as a multi-criteria decision-making (MCDP) problem [27]. The existing decision methods [26] are not suitable for our task offloading where elements are dynamically changed. However, all decision methods are efficient and effective when the environment is stable with the perfective of all elements. We propose a lightweight and multi-criteria task offloading decision method, which tackles all elements dynamically based on their current values. Furthermore, we apply similar elements pairwise comparison to the analytic hierarchy process (AHP) method [29] to obtain pairwise values. We show the pairwise value in the matrix *G*.

(16) $$\begin{array}{cc} \sum_{G=1}^{A}G= & \left[\begin{array}{cc} v_{11}\cdot fw_{11},v_{12}\cdot fw_{12},v_{13}\cdot fw_{13},v_{14}\cdot fw_{14} & \\ v_{21}\cdot fw_{21},v_{22}\cdot fw_{22},v_{23}\cdot fw_{23},v_{24}\cdot fw_{24} & \\ v_{31}\cdot fw_{31},v_{32}\cdot fw_{32},v_{33}\cdot fw_{33},v_{34}\cdot fw_{34} & \\ v_{41}\cdot fw_{41},v_{42}\cdot fw_{42},v_{43}\cdot fw_{43},v_{44}\cdot fw_{44} & \end{array}\right], \end{array}$$

(17) $$\begin{array}{cc} \omega \cdot v_{i\times k}= & 1\cdot v_{k\in K\times v_{i}\in N}=\frac{1}{v_{i\times k}}. \end{array}$$

Equation (16) describes three alternative computing nodes, i.e., $n=(k_{1},k_{2},k_{3}$) and four attributes $\omega =\{fw_{1},fw_{2}\cdots ,\omega \}$. It determines the K×N(3×4) resource matching calculation during the offloading decision. The attributes are execution time, communication time, resource availability, and deadline. The pairwise comparisons of giving elements are produced based on the normalized comparison scale on nine levels employed in matrix *G* to compute the weight of the attributes by obtaining an eigenvector ω, which is also associated to the prime eigenvalue $\lambda_{max}$. As usual, the outcome of the pairwise comparison reliability index (RI) is determined in the following way:

(18) $$\begin{array}{cc} RI= & \frac{\lambda_{max}-n}{\left(n-1\right)}, \end{array}$$

(19) $$\begin{array}{cc} RR= & \frac{RI}{(n-1)\times Random-Index}. \end{array}$$

Equation (18) shows the reliability indexes of elements, where RR is the reliability index ration of RI, whereas successive relative weights are produced by RI, the possibility of multi-criteria derive via $N_{M{\left(x_{i\times k}\right)}^{dc}}$. Where *x* is a integer value, which is equal to 1 when it has ideal fuzzy weight. Because *k* is a fascinating alternative ($k_{1},\cdots ,K$) with *N* number of tasks, it further normalizes in the following way:

(20) $$\begin{array}{c} N_{M{\left(x_{i\times k}\right)}^{dc}}=\frac{M{\left(x_{i\times k}\right)}^{dc}}{\sum_{n=1}^{4}M_{i\times k}^{2}}, \end{array}$$

Equation (20) determines the selection of the best resources among all existing resources, as *x* is any real number of *R* which explores the choices that may be criteria and alternative. Where dc is the dcth decision maker whose task has the highest rank on the computing node. Due to the dynamic environment, tasks and resource offloading have many FMCM choices, i.e., $\left\{dc_{1},\cdots ,DC\right\}$.

(21) $$\begin{array}{c} M_{w}=\omega_{v}\times N, \end{array}$$

Equation (21) stored the related weight of each element (which is fixed in advance), where $\omega_{v}$ is a weight which is already initialized above. The affirmative best solution and the aversive solutions for each decision maker can be determined dc from the weight matrix.

(22) $$\begin{array}{ccc} \; & A^{dc+}= & \\ \; & \langle \min T_{total}\times i\times k|i=1,2,3,4,k=1,2,3|N\in J^{+}\rangle , & \\ \; & \langle \min T_{total}\times i\times k|v=1,2,3,4,k=1,2,3|N\in J^{-}\rangle , & \end{array}$$

(23) $$\begin{array}{ccc} \; & A^{dc-}= & \\ \; & \langle \min T_{total}\times i\times k|v=1,2,3,4,k=1,2,3|N\in J^{-}\rangle , & \\ \; & \langle \min T_{total}\times i\times k|v=1,2,3,4,k=1,2,3|N\in J^{+}\rangle \end{array}$$

where $J^{+}$ is a positive solution of the decision maker for application objective, where $J^{-}$ is aversive (negative) solution to the objective. It is natural to consider the real time information related to the available wireless network via the network profiler; the FMCM uses the Euclidean distance matrix among all possible alternatives and it can be calculated in this way:

(24) $$\begin{array}{cc} D_{v}^{+}= & \sqrt{\sum_{v=1}^{4}{\left({\left(T_{total}\times i\times k\right)}^{+}\right)}^{2}}, \end{array}$$

(25) $$\begin{array}{cc} D_{v}^{-}= & \sqrt{\sum_{v=1}^{4}{\left({\left(T_{total}\times i\times k\right)}^{-}\right)}^{2}},\; \end{array}$$

Equation (24) determines the best choice: which solution is best for each task before offloading. Because $D_{v}^{+}$ and $D_{v}^{-}$ show the best and worst solution for each alternative, the task offloading algorithm chooses the highest rank solution $H_{v}$ solution from all alternatives, as follows:

(26) $$\begin{array}{c} H_{v}=\frac{D_{v}^{-}}{D_{v}^{+}+D_{v}^{-}}. \end{array}$$

Equation (26) finds the highest rank of each node for every task during the offloading decision.

Algorithm 2 processes the offloading mechanism for all applications onto different heterogeneous computing nodes. In step 2–4, Algorithm 2 constructs the fuzzy weight tasks and resource matrix of each application based on Equation (15). The reliability index is measured based on Equation (16). In step 6–8, the algorithm normalized the tasks index based on Equation (17). In step 9–14, Algorithm 2 constructs the decision matrix based on Equation (18) and generates the position solution and negative solution based on Equation (19). The algorithm determines each task’s best choice and worst choice for computing based on Equation (20). In the end, the optimal and ideal rank of each task onto optimal computing are constructed based on Equation (21). The output of Algorithm 2, i.e., results[i∈N,k∈K] is shown in Table 4. The offloading results of four applications, such as E-Healthcare, E-Transport, Self-Autonomous (e.g., Augmented Reality), and smart home are analyzed by FMCM with different steps. Table 4 shows that, in the result list, i.e., results[i∈N,k∈K], each task has different $T_{total}$ on different computing nodes. Therefore, these results will be passed to the system for further execution under deadlines and failure constraints.
**Algorithm 2:** Task Offloading Phase
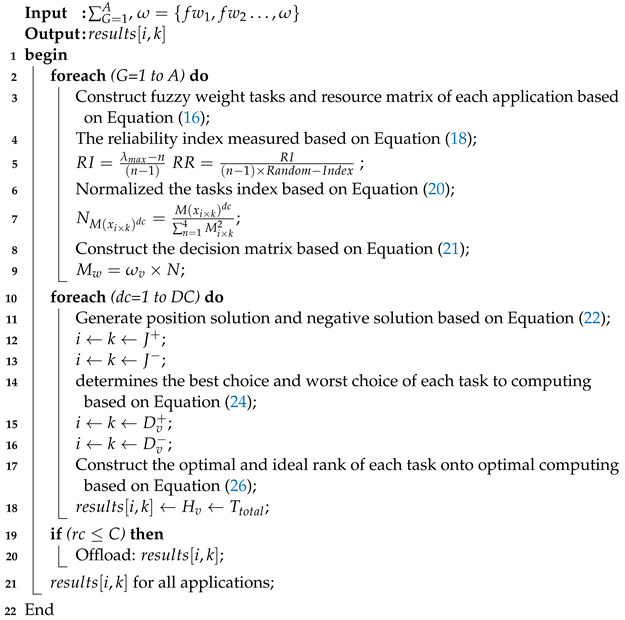


### 4.2. Topological Ordering of Tasks

Table 5 shows the topological ordering of the tasks into the system. A topological sort is a method of sorting jobs in which each vertex appears before any of the vertices reliant on it. We use a topological sort to represent the task graph using an adjacency list. The task restructuring adjacency list was ordered in this study. A period task graph topological sort algorithm is based on a topological sort algorithm. However, we compare methods by the period at a specific time. Furthermore, this approach assigns a period to all tasks using a harmonic relation. Therefore, it is based on the assumption of usage. The offloading method generated the offloading result, i.e., results[i∈N,k∈K] of tasks of all applications. Each task has different requirements, such as workload, deadline, required bandwidth, and $T_{total}$. Therefore, all tasks are sorting into some topological order, such as lateness order and deadline order. The study proposes a novel three ordering rules-based methods. In sequence-1, all tasks are sorted by descending order of their $T_{total}$ on computing nodes. In the second sequence-2, all tasks are sorted on the specific node by their deadlines. The first topological ordering of tasks based on the proposed is shown in Table 5.

The topological ordering of deadline sequence is shown in Table 6.

### 4.3. Dynamic Task Scheduling

The study devises the scheduler method Algorithm 3 for the workload sorting and execution in the system. The dynamic scheduling is the mapping process in this study which schedules all tasks onto different computing based on their given ranks or orders. The task scheduling scheme is an iterative model where the objective of each task will improve during the runtime of execution. The scheduler only handles computing node failure tasks instead of offloading failure. The study only considered the node failure tasks, which are transitory failed during their process. This study devises a task scheduling Algorithm 3 which takes a sorted list of tasks and resources as an input. We only determined the communication offloading and downloading time of tasks in the system. As we assumed, we have fixed bandwidth for the offloading and downloading task data between users and servers in the article. The study exploited the scheduler in which tasks are received in random format and stored in the queue before being scheduled in the system.

All steps of Algorithm 3 defined as below.

- The workload of all tasks must be less than the capacity of the computing nodes before scheduling in the network as defined between 2 and 5 steps.
- All tasks are sorted based on sequence-1 and sequence-2.
- If the task deadline is satisfied at any node without wasting any resource, the objective of each task is calculated based on Equation (7).
- There are three statuses of each task with different activities, such as start, i.e., status-1, and status = 2 shows progress, and status shows the finished process at a particular node as defined in 9 to 20 steps.
- If any task is incurred with transient failure, it will be added to the failure list.
- The failure list will recover tasks based on the failure aware method with their detection, retry, and failure aware strategies.
- However, due to the dynamic environment, the initial solution is not optimal. Therefore, the searching method searches for the optimal solution based on the current values of the network.
- The searching method will improve the initial solution of tasks from the neighborhood structure.
- The neighborhood structure consists of different objective functions of each task in the dynamic environment, where the scheduler will choose the best one via the linear searching method.
- The scheduler will control both the failure and dynamic environment for all tasks in the system.

**Algorithm 3:** Task Scheduling Scheme

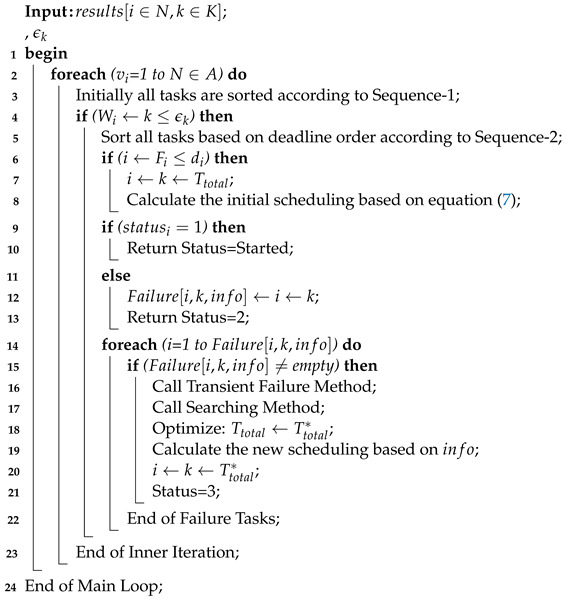



### 4.4. Solution Searching Method

The study considered the dynamic environment, where users can enter and leave the network at runtime. At different timezones, the objective function of each task could be changed. In the night timezone, the user’s traffic is low, and all requests are entertained in a better way. However, during peak time, the traffic is high and the objective function will suffer from time to time. Due to this dynamic environment, the study constructs the different solutions for each task via neighborhood structure. The study proposes a novel searching engine, i.e., Algorithm 4, which determines the best solution for each task based on offloading results in the system.

The study defines all steps of Algorithm 4 as follows.

- Initially, the study constructs the neighborhood structure of available solutions.
- The initial objective of each task is $T_{total}$.
- The initial objective of each task $T_{total}$ will be compared with another solution when a new solution is better than the existing ones, and it will return a new optimal solution, i.e., $T_{total}^{*}$.
- All the failure tasks or scheduled tasks always obtained an optimal solution, i.e., $T_{total}^{*}$ in the dynamic environment.
- The length of the search method is limited, and each solution is compared with a new solution in the non-linear way; when a new one is the better existing solution, the searching mechanism will stop searching. This way, the number of search steps will be reduced and remain optimal.

**Algorithm 4:** Searching Optimal Solution

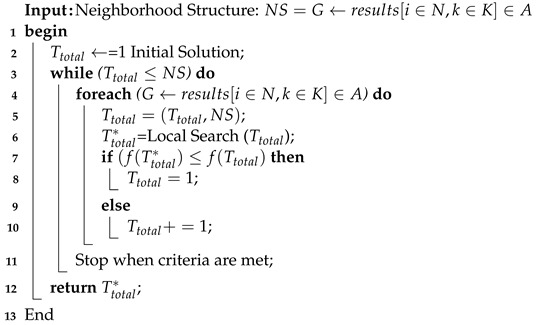



### 4.5. Transient Failure Aware Method

**Figure 2:**
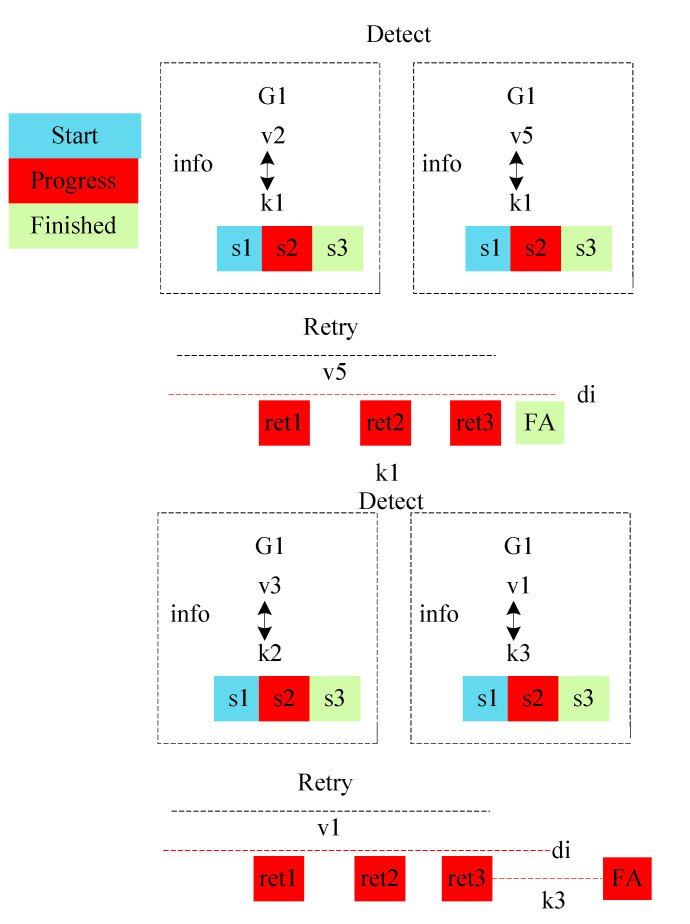
Transient Computing Node Failure Aware.

To understand the failure-aware mechanism, the study discusses a case study of the real-world practice of IoT applications. There are two types of failure in distributed computing that are often considered: communication node failure and computing node failure. However, this study considers the transient failure of computing nodes in a dynamic environment. Figure 2 illustrates the execution process of application G1 with its tasks on different computing devices. The information is the history of a task from beginning to end. Each task has three statuses: s1 shows that a task has started its execution on a particular node. s2 illustrates that a task is still in the process of execution on any node, whereas s3 ensures the execution of a task is finished successfully. The tasks $v_{2}$ and $v_{5}$ of application G1 are scheduled on $k_{1}$. Let us assume that a task $v_{5}$ failed at the computing node $k_{1}$. The detection strategy saves information on the failure of a task from the point of failure and sends it to the retry strategy. At the same time, the retry strategy tries to recover s2 status with two possible iterations left, i.e., three before the deadline, as shown in Figure 2. The task $v_{1}$ recovered with three retried operations on computing node $k_{1}$ and final failure aware (FA) policy return success status to the system. In another case, a task $v_{5}$ on computing node $k_{1}$ failed, and the retry strategy tried possible iterations. However, a task failure exceeds its deadline limit, and then the FA will mark it as a failure. The $v_{5}$ will reschedule from the scheduler from the start for execution.

Algorithm 5 handles the transient failure aware process of all applications robustly without violating their performances during execution.

The transient failure steps of Algorithm 5 are explained as below.

- Initially, the failure list of all tasks saved those tasks which have failure status during scheduling.
- The retry variable ret=0 and max-iteration (max-ite) has a limited three attempts to recover the transient failure aware process of tasks.
- The detection will return the information of tasks when they are failed on different computing nodes.
- The retry strategy will retry tasks from their point of failure with three iterations. The retry duration is only 30 s, and the gap between the first iteration and the second iteration is about 15 s.
- In the end, if the tasks are retried under their deadlines, then FA returns finished status. Otherwise, it will inform the scheduler of the tasks to be scheduled again from scratch.

### 4.6. Time Complexity of Jtos

A study mentioned above shows that the JTOS framework consists of different components, such as offloading, sequencing, searching, and scheduling. Therefore, the time complexity of JTOS is determined by various elements. The time complexity of offloading is divided into three phases: parameters, normalization, and weighting, and it is equal to O(n×n). In comparison, the time complexity of the positive ideal solution and negative ideal solution becomes n. The ranking of each task becomes *n*. Therefore, the total complexity of the offloading algorithm is equal to O(n×n). The task sequence is divided into two ordered, such as $T_{total}$ and deadline, and the time complexity becomes Olog(n×n). The $T_{total}$ ordering becomes *n* and it is the same for the deadline, which becomes *n*. Therefore, the total time complexity is equal to Olog(n×n). The searching for an optimal solution for each task to the resource is equal to n×m. *n* is several tasks, and *m* matches each resource’s iteration during searching. Furthermore, all tasks are scheduled in *n* order to the optimal solution. The total time complexity of searching and scheduling becomes (n×m+n). The final time complexity transient failure algorithm is divided into three parts: detection, retry, and failure aware event. The detection strategy finds the failure of tasks when they have failed status in *n* time, and the same for the retry method, i.e., *n*. The failure awareness is the decision scheme for all tasks, and then it is equal to *n*. Therefore, the total time complexity of transient failure is equal to n+n+n.
**Algorithm 5:** Transient Failure Aware Schemes
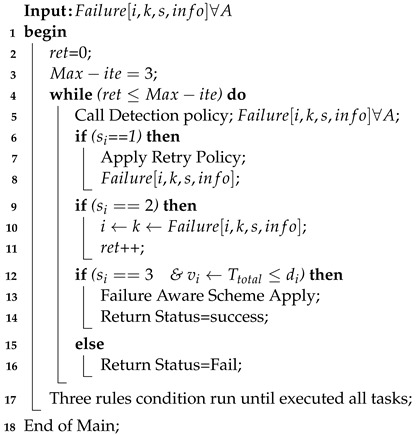


## 5. Performance Evaluation and Experimental Settings

This section evaluates the performances of the proposed algorithms on the different workloads of IoT applications in a dynamic environment. The performance evaluation consists of many sub-parts, from the parameter setting to the result in the discussion.

### 5.1. Existing Simulation Tools

Many existing simulation tools and their approaches suggested different frameworks to solve the CLIP problem in distributed computing, as shown in Table 7. These tools consist of the following parts: control plane network, framework, implementation, environment, and the problem type. The control plane is a centralized system, which handles the entire system within the system. For instance, the SDN control plane enables many BS and fog nodes and manages their management during the process. Due to offloading, these studies considered the BS, wireless access point, and Bluetooth as a network channel for offloading. The algorithm framework is most important here, and many works suggest their methods based on GA, iterative heuristic, PSO, and optimization techniques to solve the problem. The computing nodes, such as fog node and cloud, offer two kinds of servicing model based on container and virtual machines. However, recently, results are witnessed that container-type resources outperform virtual machines concerning the delay in the system.

### 5.2. Proposed Simulation Tool

The evaluation part is crucial to evaluate the performances of applications based on proposed schemes. We exploited the multi-variance analysis of variance (ANOVA) method to find out the ideal parameters of the proposed algorithm. The simulation parameters are organized in Table 8. The workload analysis of mobile cloud applications with different task types (i.e., image, text, and video) is explained in Table 9. We use a fog cloud network based on android emulators, i.e., Amazon GenyMotion, running as virtual images on the AWS product, as a service (PaaS) on virtual machines and on the desktop machine. We also implemented a cloud-based android emulator running as a virtual image on a desktop machine as software (SaaS). We constructed a virtual cloud, i.e., edge cloud, that will be scaled up and down on-demand while emulator and configurations are performed in the cloud. We designed JTOS heuristic in the JAVA language with an advanced application programming interface (API) and tested it on Intel (R) Core (TM) i5-3475 CPU @ 3.30 GHz, 10 G Memory machine. We implemented the 64 bit X86 Amazon Machine Image (AMI) mobile cloud environment with Android 7.0 nougat for mobile cloud applications. We installed mobile applications APK’s (i.e., Android Packages) on 64 bit X86 AMI. The configuration of heterogeneous computing nodes resources defined in Table 10 with their characteristics and specifications.

Table 10 defines resource specification of computing nodes with their characteristics and features.

### 5.3. Data Performance Method

We tested four benchmark mobile cloud applications; their specifications are exemplified in Table 9. We tested applications that generate data (i.e., configuration file obtained data via profiling technologies and task scheduling heuristics) of different applications via analysis of variance (ANOVA). At the same time, ANOVA is an efficient parametric technique accessible for examining algorithm-generated data of mobile cloud applications from experiments. We exploit *t*-tests and dependent and independent random variables in the one way ANOVA method to note the proposed method’s efficiency. To compute the recital of the JTOS, we exploit RPD (relative percentage deviation) statistical analysis. It evaluates the power consumption consumed by different parameters and frameworks, plus algorithm permutation throughout the parameter space of component calibration. The RPD estimation can be as demonstrated in the following Equation (27):

(27) $$\mathrm{RPD}\left(\%\right)=\frac{T_{total}-T_{total}^{*}}{T_{total}^{*}}\times 100\%,$$

where $T_{total}$ is the objective function of the article that executes the tasks via the proposed IoT tasks fog cloud architecture and JTOS algorithm. Furthermore, $T_{total}^{*}$ is the delay optimal and scheduling in the proposed architecture in the distributed computing environment.

### 5.4. Baseline Approaches

The study implemented the recently published article methods to compare the performance of the proposed JTOS framework based on different components. Recently, two computing models, virtual machines and container-based services, have been widely used to run various IoT applications. However, many additional features, such as service start time, pre-allocation, and post-allocation of resources, have utilization and delay effects on both applications and the system. The following research methods are assumed as baseline approaches in the experimental part.

(1) **Baseline 1:** The experiment environment implemented existing method frameworks [1,2,3,4,5,6,7,8,9,10] that are considered dynamic situations during offloading and resource allocation. They suggested joint offloading and GA and PSO-aware resource allocation methods to CILP problems with the fixed environment (e.g., where users entered and left at different timezone). They considered a virtual machine-based fog cloud network and SDN controller with different base stations and wireless access points.
(2) **Baseline 2:** The experiment environment implemented existing method frameworks [11,14,19,22,39] that are considered dynamic situation during offloading and resource allocation. They suggested joint offloading and iterative and search-aware greedy heuristics-based resource allocation methods to CILP problems with the fixed environment (e.g., where users entered and left at different timezone). They considered a docker container-based fog cloud network and SDN controller with different base stations and wireless access points.

### 5.5. Sdn Fog Nodes Offloading Scenario

#### Sdn Controller Configuration

NetSim [40] is a widely used simulation modeling for the SDN control plane to handle distributed fog and cloud computing via different base stations. This study extended the SDN class control plane and base-station classes as an abstract class from the NetSim model for the experiment purpose in the proposed work.

The SDN control plane monitors the channel capacity and resource of computing nodes. The SDN control plane is a management manager that helps offload the engine to reduce the total delay of applications. However, in different timezones, the uncertainty of applications could be added due to traffic and load on nodes. The study considered the following assumptions during offloading for all tasks.

1. Each task $v_{i}$ is only finished and its execution or fail once it is assigned to any node.
2. Each task has a different total delay in a different timezone.
3. The study only considered the node failure and ignored the base-station failure.
4. The task migration and pre-emption are not allowed.
5. The dynamic environment only considered enter and leaving requests in the network. It is different from mobility, and mobility is not considered in this study.
6. The failure tasks reschedule from scratch if they completely failed on their computing nodes.
7. The roundtrip time between user and base station and base station to computing nodes is fixed in the same timezone. However, in a different timezone, there would be a different network roundtrip during offloading.

**Figure 3:**
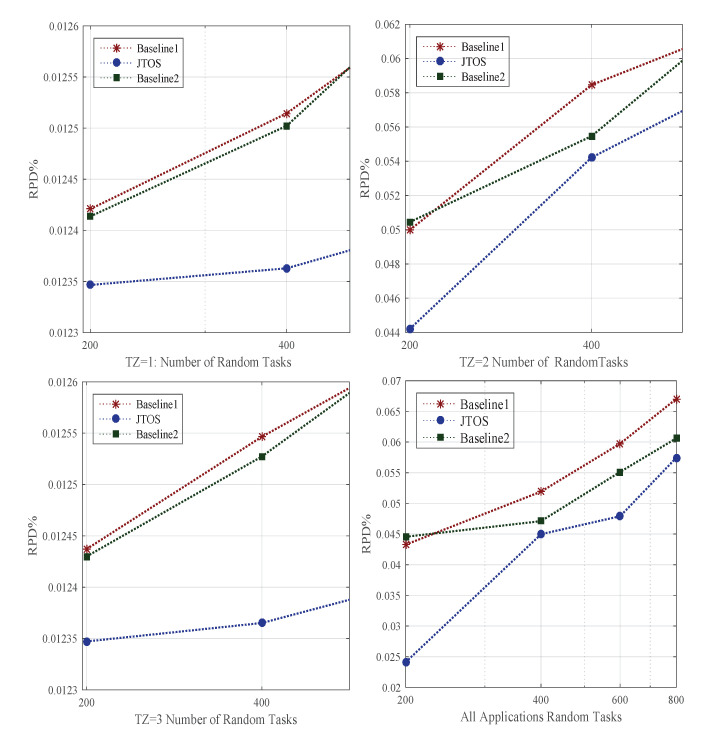
Offloading Performance In Different Timezones.

The offloading of tasks in different timezones has an impact on the objective of each application. Whereas in a dynamic environment, users with different applications can enter and leave at any time. However, the traffic of users becomes dense from 8 am to 10 pm. Therefore, in the simulation, the timezone is divided into three zones, such as TZ = 1, i.e., 12 am to 8 am; this time, the traffic is more minor, and total delay (communication delay and computation delay) becomes less. Whereas TZ=2, i.e., 8 am to 4 pm, the traffic becomes very high, applications’ objective function has a huge impact on their performances. In the final timezone TZ=3, i.e., 4 pm to 12 am, the traffic becomes less than daytime peak time. In the joint offloading and allocation optimization problem, existing studies only consider the random and linear offloading without considering the timezone. Furthermore, they offload tasks based on task size and required CPU requirements. However, they did not consider the deadline and total delay of applications in their offloading scheme. Figure 3 shows FMCM in JTOS gained lower delay of application with random tasks as compared to all baseline approaches. The study evaluated the offloading of all applications at different timezones with JTOS with a different number of arbitrary tasks. Hence it is proved that the multi-criteria offloading in a dynamic environment with different timezones outperforms all conventional offloading methods in terms of RPD%. In the following way, the proposed FMCM works better than the existing offloading method: (1) All existing offloading methods in baseline 1 and baseline 2 just focused on compute-intensive or data-intensive task offloading in the full offloading scheme. Whereas, in full offloading, all tasks were offloaded in the associated external server for execution. The task offloading scheme could not meet the QoS requirement of applications, such as deadline and objective function based on data size. (2) The study proposed a fuzzy multi-criteria method that offloads all tasks based on data size, execution time, total delay, and deadline of each application in a different timezone. Different timezones have different offloading results; FMCM in JTOS always adopts the environment and makes an offloading decision based on the current values in the system.

### 5.6. Proposed Task Scheduling Performance against State-of-the-Art Approaches

**Figure 4:**
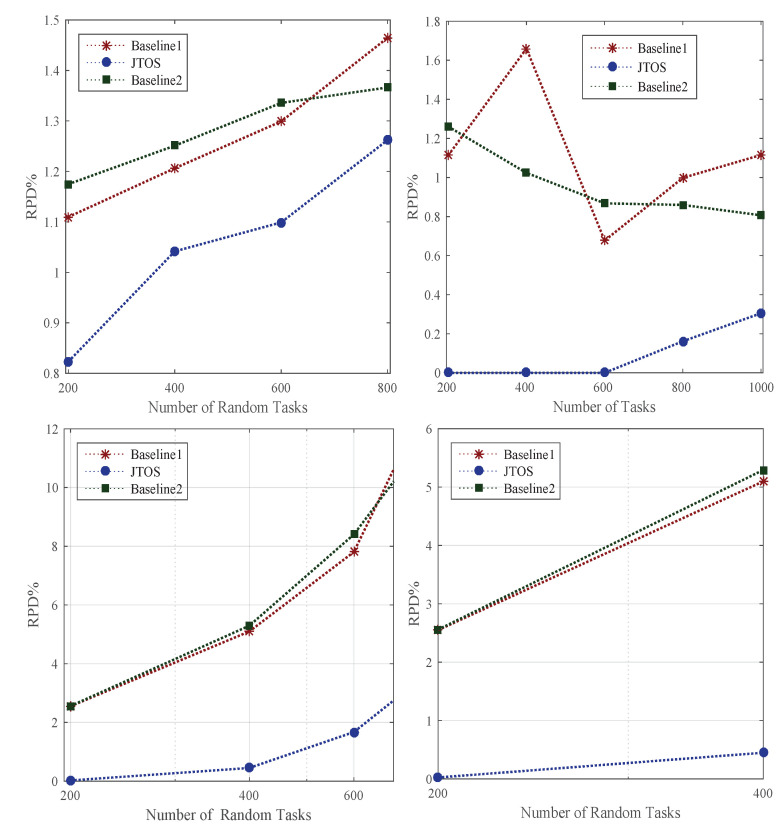
Scheduling Performance At Different Timezone.

Scheduling (e.g., resource allocation) is always a challenging problem in the dynamic environment. Whereas ordering of tasks, i.e., ranks, searching for particular resources, and transient failure often happen in the background. Due to different requirements of tasks in size, deadline, and additional computing speed of nodes, they are assigning tasks appropriately based on their ranks. For instance, $v_{1}$ has a small deadline compared to $v_{2}$, which has both a large size and deadline. Therefore, in this case, the scheduler should not assign them to power machines for their execution. In this way, a lot of resources are wasted. We implemented the existing GA, PSO, and HEFT-based methods as existing studies used in their resource allocation model and evaluated all applications’ performance in terms of RPD% in the dynamic environment. Figure 4 shows the performance of all random tasks of different applications at different timezones onto heterogeneous computing nodes. Initially, all algorithms rank to each task before scheduling in the network, then search the appropriate resource for the execution of every task. Different searches such as liner search informed search and bi-searched are implemented as baseline approaches in the simulation tool. Figure 4 illustrates that JTOS outperforms all existing resource allocation policies in terms of RPD% of applications in different timezones with their characteristics.

These are the reasons why JTOS outperforms all existing baseline approaches: (1) The first reason is that they exploited a simple sorting strategy, i.e., all tasks are ranked based on their size. However, this way, they will violence their deadline during scheduling. (2) The second reason, all tasks are sorted based on the available resource of machines. However, in this way, the slack time gap between tasks leads to lateness for each task. (3) All existing resource allocation schemes were used for exploration and exploitation searching during scheduling for all applications. However, due to the ample space of candidate searching, the scheduling will face a lot of overhead during the assignment of tasks. (4) They implemented failure aware strategy and recalled backup or checkpointing strategy until and unless tasks are recovered from the point of failure. However, this way, resource and recovery time become very high. Therefore, scheduling in JTOS ranks all tasks based on size, execution time, deadline, and total delay before scheduling. The initial scheduling maps all tasks based on sorting order. Then, the failure of tasks reschedules from the failure aware strategy with minimum recovery time compared to existing studies. In the dynamic environment at different timezones, JTOS outperforms for all applications in terms of RPD%.

### 5.7. Proposed Failure Aware Technique Performance against State-of-the-Art Approaches

**Figure 5:**
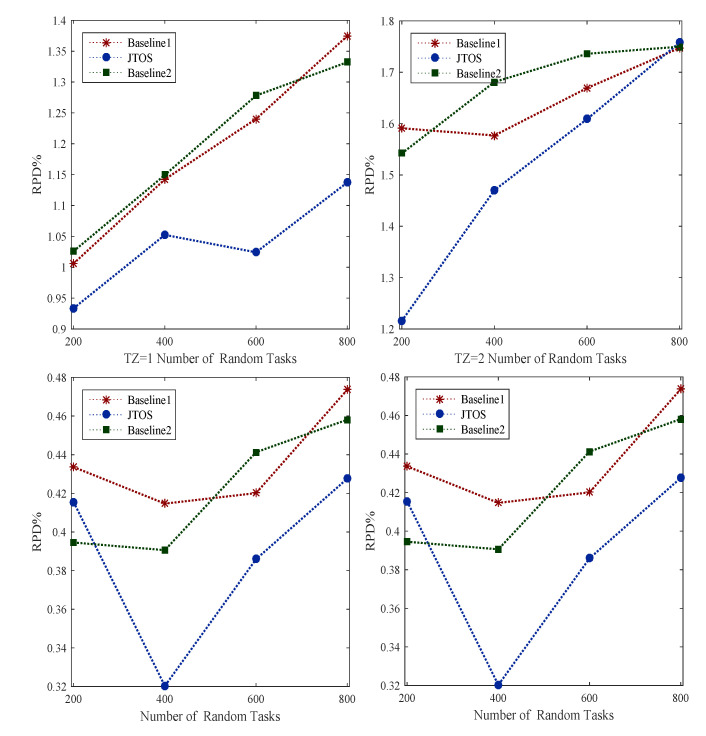
Failure Aware Performances At Different Timezones.

The failure of tasks in the dynamic environment often occurs at different timezone due to intermittent changes in network and computing nodes. We implemented all existing strategies of fault-tolerant in distributed computing. Baseline 1 and baseline 2 are implemented checkpointing, backup recovery, runtime backup recovery, and node failure. However, these policies cannot apply to the transient failure of tasks in a dynamic environment. The transient failure is a temporary failure that can recover under task deadlines. The transient failure methods recover the failure of tasks on the same node instead of transferring to another node. For instance, $v_{1}$ is failed due to computing node $k_{1}$, and it is a transient failure; it will recover soon after some instants under the task deadline. It requires some iterations for recovery. However, the recovery will complete under its deadline. The study implemented all existing failure methods and evaluated the performance of all applications in terms of RPD%. Figure 5 shows the transient failure aware schemes in JTOS handled all types of errors with detection scheme, retry scheme, and failure aware scheme and gained good performance as compared to all existing baseline approaches. However, still, the failure ratio is high because many transient failures need to be recovered with deadlines. For instance, communication failure, application failure, switches failure, and busy failure still need to be recovered in the transient failure aware methods.

However, Figure 5 shows that the failure ratio is still high in the dynamic environment because all methods did not consider the failure of tasks due to the communication node in a different timezone. The resource fluctuation could occur in other computing during peak hours because of many requests generated by users. Therefore, it is necessary to measure the performances of the system in a different timezone. The proposed JTOS algorithm offers timezone-aware scheduling without degrading the quality of tasks at an additional time.

### 5.8. Rescheduling and Searching Delay Performance against State-of-the-Art Approaches

**Figure 6:**
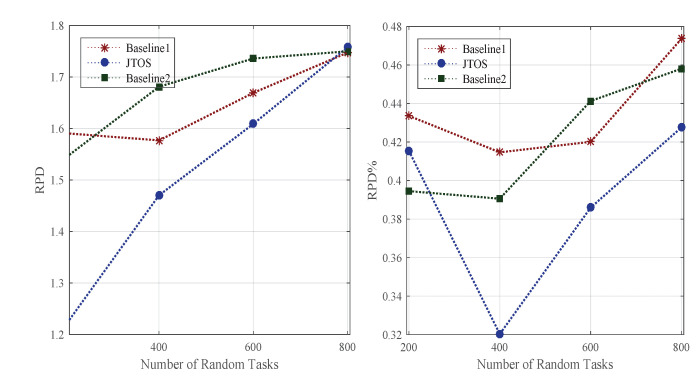
Solution Searching Aware Performances At Different Timezones.

The searching for a new resource after the failure of a task is necessary for the scheduling method. For instance, if a task $v_{1}$ is currently executing on computing node $k_{1}$, and if a task cannot recover, it will schedule from scratch with a new resource, i.e., $k_{2}\in K$. It is important, and a new computing node must execute the failure of tasks without wasting a lot of resources. The study proposed a searching mechanism for failure of tasks based on tasks’ objective function and executing them under their deadline without violence of their performances. Figure 6 shows that all failed tasks are executed under their deadlines with minimum loss of generosity. Whereas, these methods will still improve when the communication and computing failure of tasks are noted in the dynamic environment for execution. However, the searching mechanism in JTOS for the failure of tasks still work better than existing methods in terms of applications of RPD%.

## 6. Conclusions and Future Work

This study formulated the joint offloading and scheduling CILP problem for IoT applications in the distributed fog cloud network. The proposed JTOS algorithm framework executed all tasks with different components under deadline and failure-aware constraints. The performance evaluation showed that JTOS outperforms all existing joint offloading and scheduling problems in the dynamic environment. The results discussed showed that the proposed work executed the successful industrial automation applications on the collaborative fog cloud network. All the results differed, and the proposed work minimized the overall delays compared to existing studies by 50% in work. However, there are a lot of limitations in the proposed schemes to be improved in future work. The JTOS does not support mobility-aware and location-aware services for IoT applications. This work still suffers from security issues in the fog cloud network. Application failure, communication failure, and node failure are standard transient errors in the network. However, this study only considered momentary node failure for IoT applications.

This study formulated the joint offloading and scheduling CILP problem for IoT applications in the distributed fog cloud network. The proposed JTOS algorithm framework executed all tasks with different components under deadline and failure-aware constraints. The performance evaluation showed that JTOS outperforms all existing joint offloading and scheduling problems in the dynamic environment.

The researched technologies have great potential for application within the Industry 4.0/5.0 concept. For the development of predictive maintenance in the industry, the use of IoT and fog cloud will be key.

In future research, the author’s collective will deal with the design of a suitable architecture for the needs of Industry 4.0/5.0. It will also be a key element of the Operator 4.0/5.0 concept.

## Figures and Tables

**Table 1:** Existing offloading methods.

Research	Parameters	Decision	Profiling	Environment	Problem	Objective
[1]	Single Para.	Static	Network	Fixed	ILP	Min.Energy
[2]	Single Para.	Static	Program	Fixed	LP	Min.Energy
[3]	Single Para.	Static	REST API	Fixed	MLP	Min Computation
[4]	Two Para.	Dynamic	RPC	Adaptive	Concave	Max.Utilization
[5]	Multi-Para.	Dynamic	Monitoring	Adaptive	Quadratic	Max. Throughput
[6,7]	Multi-Para.	Dynamic	Resource	Adaptive	Integer-Constraints	Min.Delay
[8,9,10]	Multi-Para.	Dynamic	Monitoring	Adaptive	Quadratic	Min.Energy
[8,11]	Multi-Para	Hybrid	Monitoring	Mobility	ILP	Min Rent
[12,13,14]	Many-Para.	Hybrid	SDN-Controller	Mobility	ILP	Min.Cost
[15,16,17]	Many-Para.	Hybrid	SDN-Controller	Mobility	LP	Min.Budget
[18,19]	Many-Para.	Hybrid	OS	Mobility	ILP	Min.renting cost
[20,21]	Many-Para.	Hybrid	OS	Mobility	ILP	Min.Energy
[1,3,5,8,11]	GA	Many	Fog-Cloud	VMs	Olog(m×n)	Offloading
[2,4,6,10,12]	PSO	Many	Edge/Cloud	VMs	$Olog{\left(m\times n\right)}^{2}$	Joint-ORA
[13,14,15,16,17,18,19]	Heuristics	Many	Edge	VMs	M×N×N	Offloading
[20,21,22,23,25,26]	DRL	Many	Edge-Cloud	Containers	M×M×N	Scheduling

**Table 2:** Mathematical notation.

Notation	Description
*A*	Number of all IoT applications
*G*	The *G*th application of *A*
*N*	Number of tasks of application *G*
$v_{i}$	*i*th independent task
$v_{i}↔b$	Round-trip delay between $v_{i}$ and *b*
*B*	Set of base stations *b*
$y_{ib}$	The coverage base stations
b↔k	The Round-trip delay between *b* and *k*
$T_{i}^{net}$	Total network delay for task $v_{i}$
*M*	Set of computing nodes
*k*	*k*th computing nodes of *M*
$\epsilon_{k}$	Resource capacity of computing node kth *k*th
$\zeta_{j}$	The speed capability of *j*th of *k*th computing node
$W_{i}$	The requested workload of task $v_{i}$
$W_{i}^{\prime }$	The result of task $v_{i}$
$d_{i}$	The deadline of task $v_{i}$
$T_{i}^{e}$	The execution time of task $v_{i}$
$x_{ij}$	The assignment of task $v_{i}$ on $k_{j}$ cloud
$F_{i}$	The finish time of task $v_{i}$
Td	The lateness of the $v_{i}$
*C*	The channel capacity
$B_{w}$	The bandwidth of the channel in hertz
*S*	Signal power over the bandwidth
$N_{s}$	Interference over the bandwidth
$\frac{S}{N_{s}}$	The signal-to-noise ratio

**Table 3:** Scale & definition.

Definition	Strength of Importance
Uniformly	1
Fairly important	3
Robustly important	5
Very robustly important	7
Extremely important	9
Intermediary	2 4 6 8

**Table 4:** $T_{total}$ Delay Result of Tasks on Heterogeneous Computing Nodes.

$G_{1}$	Fog Nodes			Cloud Node
	$k_{1}$	$k_{2}$	$k_{3}$	$k_{4}$
$v_{1}$	30	20	10	50
$v_{2}$	27	28	47	70
$v_{3}$	51	26	29	80
$v_{4}$	50	47	71	29
$v_{5}$	24	57	56	77
$v_{6}$	35	26	16	34.5
$G_{2}$	Fog Nodes			Cloud Node
	$k_{1}$	$k_{2}$	$k_{3}$	$k_{4}$
$v_{1}$	41	31	51	66
$v_{2}$	47	30	31	86
$v_{3}$	36	29	27	21
$v_{4}$	41	37	71	33
$v_{5}$	33	30	18	54
$v_{6}$	19	26	49	59
$G_{3}$	Fog Nodes			Cloud Node
	$k_{1}$	$k_{2}$	$k_{3}$	$k_{4}$
$v_{1}$	17	31	15	100
$v_{2}$	34	41	17	120
$v_{3}$	48	25	55	90
$v_{4}$	61	66	86	43
$v_{5}$	86	76	52	48
$v_{6}$	12	65	19	39
$G_{4}$	Fog Nodes			Cloud Node
	$k_{1}$	$k_{2}$	$k_{3}$	$k_{4}$
$v_{1}$	21	45	15	100
$v_{2}$	56	90	50	46
$v_{3}$	89	125	60	56
$v_{4}$	20	66	46	130
$v_{5}$	32	26	50	200
$v_{6}$	200	285	240	100

**Table 5:** Sequence-1: Sorting Tasks by Their Descending Order of Resource $T_{total}$.

	Fog Nodes			Cloud Node
Application	$k_{1}$	$k_{2}$	$k_{3}$	$k_{4}$
$G_{1}$	$v_{2},v_{5}$	$v_{3}$	$v_{1},v_{6}$	$v_{4}$
$G_{2}$	$v_{6}$	$v_{2},v_{1}$	$v_{5}$	$v_{3},v_{4}$
$G_{3}$	$v_{6}$	$v_{3}$	$v_{2}$	$v_{4},v_{5}$
$G_{4}$	$v_{1},v_{4}$	$v_{5}$	$v_{1}$	$v_{3},v_{6}$

**Table 6:** Sequence-2: Sorting Tasks by Their Descending Order of Resource $d_{i}$.

	Fog Nodes			Cloud Node
Application	$k_{1}$	$k_{2}$	$k_{3}$	$k_{4}$
$G_{1}$	$v_{5},v_{2}$	$v_{3}$	$v_{6},v_{1}$	$v_{4}$
$G_{2}$	$v_{6}$	$v_{1},v_{2}$	$v_{5}$	$v_{4},v_{3}$
$G_{3}$	$v_{6}$	$v_{3}$	$v_{2}$	$v_{4},v_{5}$
$G_{4}$	$v_{1},v_{4}$	$v_{5}$	$v_{1}$	$v_{6},v_{3}$

**Table 7:** Existing SDN Fog Cloud Simulation Tool.

Tool	Control Plane	Network	Framework	Implementation	Environment	Problem
Edge-Fog [30]	SDN	BSs	GA	Fog VMs	Dynamic	CILP
Fog-Torch [31]	SDN	Wireless	HEFT	Fog VMs	Dynamic	CILP
Fog-TorchII [32]	Agent	BSs	SA	Edge VMs	Dynamic	CILP
iFogSim [33]	Agent	BSs	Iterative	Edge VMs	Dynamic	CILP
FogDirSim [34]	Controller	BSs	Heuristic	Edge VMs	Dynamic	CILP
FogNetSim++ [35]	Controller	BSs	Meta-Heuristic	Fog VMs	Dynamic	CILP
FogWorkFlow- Sim [36]	Master Node	Wireless	Searching	Fog VMs	Dynamic	CILP
YAFS [37]	Hadoop	Wireless	Min-Max	Fog Container	Dynamic	CILP
FogDirMime [38]	Handler	Link	PSO	Fog Container	Dynamic	CILP
fogbus [39]	Monitor	Nodes	MET	Fog VMs	Dynamic	CILP
MobFogSim [39]	Profiler	Wireless	MCT	Fog Container	Dynamic	CILP

**Table 8:** Simulation Parameters.

Simulation Parameters	Values
Windows OS	Linux Amazon GenyMotion (virtual box)
Centos 7 Runtime	X86-64-bit AMI
Languages	JAVA, XML, Python
Android Phone	Google Nexus 4, 7, and S
Experiment Repetition	160 times
Simulation Duration	12 h
Simulation Monitoring	Every 1 h
Evaluation Method	ANOVA Single and Multi-Factor
Amazon On Demand Service	EC2 t3
Android Operating System	GenyMotion
Application interface	Desktop, Cloud or Mobile Applications:
**Task Offloading Attributes**	**Weights**
*C*	5GB
*B*	0.130
$\frac{S}{N}$	0.062
RR	0.052
$v_{i}↔b$	0.1∼100 ms
b↔k	0.05∼50 ms
ω	{0.1, 0.4, 0.3, 0.5}
TZ=1	12 am to 8 am
TZ=2	8 am to 4 pm
TZ=3	4 pm to 12 am

**Table 9:** Workload of IoT Applications.

Workload	$W_{i}$ (MB)	$W_{i}^{\prime }$ (MB)	Image Tasks	Video Tasks	Text Tasks	*N*
$G_{1}$	825	5.2	100	100	300	500
$G_{2}$	631	6.3	150	200	350	700
$G_{3}$	645	7.4	100	200	500	800
$G_{4}$	755	8.7	200	200	600	1000

**Table 10:** Heterogenous Fog Cloud Nodes Resource Specification.

Resources	Fog Clouds			Public Cloud
	$k_{1}$	$k_{2}$	$k_{3}$	$k_{4}$
$\zeta_{k}$	Small 2 V CPU	Medium 4 V CPU	Large 8 V CPU	Extra Large 24 V CPU
CORE	1	1	2	4
$\epsilon_{k}$	500 GB	1000 GB	1500 GB	3000 GB
Run−Time	X86	X86	X86	X86

## Data Availability

All the experimental data are generated at the local institution servers. Therefore, it cannot be made publicly available for other researchers.

## References

[1] De D., Mukherjee A., Roy D.G. (2020). Power and Delay Efficient Multilevel Offloading Strategies for Mobile Cloud Computing. Wirel. Pers. Commun..

[2] Shahryari O.K., Pedram H., Khajehvand V., TakhtFooladi M.D. (2020). Energy-Efficient and Delay-Guaranteed Computation Offloading for Fog-Based IoT Networks. Comput. Netw..

[3] Aburukba R.O., AliKarrar M., Landolsi T., El-Fakih K. (2020). Scheduling Internet of Things requests to minimize latency in hybrid Fog–Cloud? computing. Future Gener. Comput. Syst..

[4] Lin C., Han G., Qi X., Guizani M., Shu L. (2020). A Distributed Mobile Fog Computing Scheme for Mobile Delay-Sensitive Applications in SDN-Enabled Vehicular Networks. IEEE Trans. Veh. Technol..

[5] Fan Q., Ansari N. (2018). Application aware workload allocation for edge computing-based IoT. IEEE Internet Things J..

[6] Kavitha B., Vallikannu R., Sankaran K.S. (2020). Delay-aware concurrent data management method for IoT collaborative mobile edge computing environment. Microprocess. Microsystems.

[7] Chanyour T., El Ghmary M., Hmimz Y., Cherkaoui Malki M.O. (2019). Energy-efficient and delay-aware multitask offloading for mobile edge computing networks. Trans. Emerg. Telecommun. Technol..

[8] Chamola V., Tham C.K., Gurunarayanan S., Ansari N. (2020). An optimal delay aware task assignment scheme for wireless SDN networked edge cloudlets. Future Gener. Comput. Syst..

[9] Roy P., Sarker S., Razzaque M.A., Hassan M.M., AlQahtani S.A., Aloi G., Fortino G. (2020). AI-enabled mobile multimedia service instance placement scheme in mobile edge computing. Comput. Netw..

[10] Gu X., Zhang G., Cao Y. (2020). Cooperative mobile edge computing-cloud computing in Internet of vehicle: Architecture and energy-efficient workload allocation. Trans. Emerg. Telecommun. Technol..

[11] Zhang L., Ansari N. (2020). Latency-aware IoT Service Provisioning in UAV-aided Mobile Edge Computing Networks. IEEE Internet Things J..

[12] Xia Q., Lou Z., Xu W., Xu Z. (2020). Near-Optimal and Learning-Driven Task Offloading in a 5G Multi-Cell Mobile Edge Cloud. Comput. Netw..

[13] Abbasi M., Pasand E.M., Khosravi M.R. (2020). Workload Allocation in IoT-Fog-Cloud Architecture Using a Multi-Objective Genetic Algorithm. J. Grid Comput..

[14] Ying Wah T., Gopal Raj R., Lakhan A. (2020). A novel cost-efficient framework for critical heartbeat task scheduling using the Internet of medical things in a fog cloud system. Sensors.

[15] Arikumar K., Natarajan V. (2021). FIoT: A QoS-Aware Fog-IoT Framework to Minimize Latency in IoT Applications via Fog Offloading. Evolution in Computational Intelligence.

[16] Siasi N., Jasim M., Aldalbahi A., Ghani N. (2020). Delay-Aware SFC Provisioning in Hybrid Fog-Cloud Computing Architectures. IEEE Access.

[17] Naha R.K., Garg S., Chan A., Battula S.K. (2020). Deadline-based dynamic resource allocation and provisioning algorithms in fog-cloud environment. Future Gener. Comput. Syst..

[18] Lakhan A., Li X. (2020). Transient fault aware application partitioning computational offloading algorithm in microservices based mobile cloudlet networks. Computing.

[19] Lakhan A., Khan F.A., Abbasi Q.H. Dynamic Content and Failure Aware Task Offloading in Heterogeneous Mobile Cloud Networks. Proceedings of the 2019 International Conference on Advances in the Emerging Computing Technologies (AECT).

[20] Lakhan A., Sajnani D.K., Tahir M., Aamir M., Lodhi R. (2018). Delay sensitive application partitioning and task scheduling in mobile edge cloud prototyping. Proceedings of the International Conference on 5G for Ubiquitous Connectivity.

[21] Mahesar A.R., Lakhan A., Sajnani D.K., Jamali I.A. (2018). Hybrid delay optimization and workload assignment in mobile edge cloud networks. Open Access Libr. J..

[22] Lakhan A., Xiaoping L. Energy aware dynamic workflow application partitioning and task scheduling in heterogeneous mobile cloud network. Proceedings of the 2018 International Conference on Cloud Computing, Big Data and Blockchain (ICCBB).

[23] Lakhan A., Li X. Content Aware Task Scheduling Framework for Mobile Workflow Applications in Heterogeneous Mobile-Edge-Cloud Paradigms: CATSA Framework. Proceedings of the 2019 IEEE International Conference on Parallel & Distributed Processing with Applications, Big Data & Cloud Computing, Sustainable Computing & Communications, Social Computing & Networking (ISPA/BDCloud/SocialCom/SustainCom).

[24] Pham Q.V., Fang F., Ha V.N., Piran M.J., Le M., Le L.B., Hwang W.J., Ding Z. (2020). A survey of multi-access edge computing in 5G and beyond: Fundamentals, technology integration, and state-of-the-art. IEEE Access.

[25] Sajnani D.K., Mahesar A.R., Lakhan A., Jamali I.A. (2018). Latency Aware and Service Delay with Task Scheduling in Mobile Edge Computing. Commun. Netw..

[26] Ma X., Wang S., Zhang S., Yang P., Lin C., Shen X.S. (2019). Cost-efficient resource provisioning for dynamic requests in cloud assisted mobile edge computing. IEEE Trans. Cloud Comput..

[27] Zhang J., Xia W., Yan F., Shen L. (2018). Joint computation offloading and resource allocation optimization in heterogeneous networks with mobile edge computing. IEEE Access.

[28] Hossain M.D., Sultana T., Nguyen V., Nguyen T.D., Huynh L.N., Huh E.N. (2020). Fuzzy Based Collaborative Task Offloading Scheme in the Densely Deployed Small-Cell Networks with Multi-Access Edge Computing. Appl. Sci..

[29] Dab B., Aitsaadi N., Langar R. A novel joint offloading and resource allocation scheme for mobile edge computing. Proceedings of the 2019 16th IEEE Annual Consumer Communications & Networking Conference (CCNC).

[30] Mohan N., Kangasharju J. Edge-Fog cloud: A distributed cloud for Internet of Things computations. Proceedings of the 2016 Cloudification of the Internet of Things (CIoT).

[31] Brogi A., Forti S. (2017). QoS-aware deployment of IoT applications through the fog. IEEE Internet Things J..

[32] Brogi A., Forti S., Ibrahim A. How to best deploy your fog applications, probably. Proceedings of the 2017 IEEE 1st International Conference on Fog and Edge Computing (ICFEC).

[33] Gupta H., Vahid Dastjerdi A., Ghosh S.K., Buyya R. (2017). iFogSim: A toolkit for modeling and simulation of resource management techniques in the Internet of Things, Edge and Fog computing environments. Softw. Pract. Exp..

[34] Forti S., Pagiaro A., Brogi A. (2020). Simulating FogDirector Application Management. Simul. Model. Pract. Theory.

[35] Qayyum T., Malik A.W., Khattak M.A.K., Khalid O., Khan S.U. (2018). FogNetSim++: A toolkit for modeling and simulation of distributed fog environment. IEEE Access.

[36] Liu X., Fan L., Xu J., Li X., Gong L., Grundy J., Yang Y. FogWorkflowSim: An automated simulation toolkit for workflow performance evaluation in fog computing. Proceedings of the 2019 34th IEEE/ACM International Conference on Automated Software Engineering (ASE).

[37] Lera I., Guerrero C., Juiz C. (2019). YAFS: A simulator for IoT scenarios in fog computing. IEEE Access.

[38] Forti S., Ibrahim A., Brogi A. (2019). Mimicking FogDirector application management. SICS Softw.-Intensive -Cyber-Phys. Syst..

[39] Tuli S., Mahmud R., Tuli S., Buyya R. (2019). Fogbus: A blockchain-based lightweight framework for edge and fog computing. J. Syst. Softw..

[40] Calvo-Fullana M., Mox D., Pyattaev A., Fink J., Kumar V., Ribeiro A. (2021). ROS-NetSim: A Framework for the Integration of Robotic and Network Simulators. IEEE Robot. Autom. Lett..

